# Patient powered research: an approach to building capacity for a hardly reached patient population to engage in cancer research

**DOI:** 10.1186/s40900-021-00317-7

**Published:** 2021-10-26

**Authors:** Marjory Charlot, Kelsi Carolan, Cyrena Gawuga, Elmer Freeman, Linda Sprague Martinez

**Affiliations:** 1 Division of Oncology, University of North Carolina School of Medicine, Houpt Physicians Office Building, 170 Manning Drive, 3rd Floor, Chapel Hill, NC 27599 USA; 2 University of North Carolina Lineberger Comprehensive Cancer Center, Chapel Hill, NC USA; 3 University of Connecticut School of Social Work, Hartford, CT USA; 4 Boston University School of Social Work, Boston, MA USA; 5 Macro Department, Boston University School of Social Work, Boston, MA USA; 6 Center for Community Health Education Research and Service, Inc., Boston, MA USA

**Keywords:** Patient engagement, Patient advisory council, Cancer research, Patients of color, Black patients

## Abstract

**Background:**

Participating in clinical trials is a metric of high-quality cancer care and improves survival. However, Black individuals with cancer are less likely to be enrolled in clinical trials and experience a disproportionate burden of cancer mortality compared to Whites. Patient-engaged research is one potential strategy to address racial inequities in clinical trials, but little is known about best practices for engaging Black individuals and communities impacted by cancer in research partnerships.

**Methods:**

We used a community engaged research approach to establish a patient advisory council (PAC) representative of the patient population served by a safety net hospital cancer center. We outline the process of establishing the PAC and the lessons learned.

**Results:**

The inaugural PAC included 7 members representative of the cancer center’s patient demographics. PAC members developed a patient centered vision, mission and action plan. PAC and community-academic research partners experienced the transformative power of centering the lived experiences of patients of color to promote health equity in cancer research.

**Conclusion:**

Establishing a patient advisory council at a safety net hospital cancer care center provided a platform for engaging a hardly reached population in patient centered research.

**Supplementary Information:**

The online version contains supplementary material available at 10.1186/s40900-021-00317-7.

## Plain English summary

Participating in clinical trials is an important measure of high-quality cancer care and improves survival. However, Black individuals with cancer are less likely to participate in clinical trials and are more likely to die from cancer compared to Whites. Including Black patients as research partners is one way to improve racial equity in clinical trial participation. We established a patient advisory council (PAC) including patients and caregivers with similar racial demographics as the patients receiving care at a safety net hospital cancer center. PAC members partnered with the research team to develop a vision, mission, and action plan to improve research participation among patients of color. PAC members used their lived experiences and training from the research team to help develop a strategy to improve representation of patients of color in cancer research. This paper is focused on the PAC development process.

**Supplementary Information:**

The online version contains supplementary material available at 10.1186/s40900-021-00317-7.

## Background

Black persons in the U.S. experience a disproportionate burden of cancer morbidity and mortality and are far less likely to be enrolled in cancer clinical trials compared to whites [1–4]. Participation in cancer clinical trials provides access to cutting edge treatments and improves quality of care and survival [5, 6]. Racial inequities in the opportunity to participate in clinical trials arise from systemic and interpersonal racism exhibited by the mistreatment and exploitation of Black individuals in clinical research and medical care, negative stereotypes and bias against Black patients, and poor provider communication in racially discordant dyads [7–12].

One potential strategy to address racial inequities in clinical trials is to engage Black patients, caregivers, and community members as research partners [13–16]. A key principle of the Public Health Critical Race Praxis, an antiracism framework grounded in critical race theory, is to center the voices of marginalized populations in addressing racial health inequities [17, 18]. Patient-engaged research, a form of participatory research, disrupts traditional power dynamics between ‘researchers’ and the ‘researched’ while centering patients’ lived experiences with illness and navigating the health care system [16, 19]. Patient-engaged research can promote equity by including diverse stakeholders through patient advisory councils (PACs) [15, 19–23]. PACs have been shown to be successful in improving participant recruitment and retention in clinical research [20], but a key challenge is engaging diverse members reflective of a health system’s population [24–26]. So called “hard to reach” populations, for example patients of color, are typically underrepresented in PACs [25, 27–29].

Community engaged research, another participatory research approach, provides a model for engaging those traditionally left out of the research process and involves researchers and community stakeholders working collaboratively [30–33]. Engaging community stakeholders, including patients and their families, is critical to understanding the ways in which life circumstances, and socio-environmental conditions influence health and access to health care [34]. Community engagement can provide researchers with a more nuanced understanding of patient experiences, priorities and community conditions, while simultaneously empowering communities [21]. Co-learning and empowerment facilitate the diffusion of knowledge, skills, and power among research partners which can yield novel interventions that are culturally appropriate, as well as community specific, and thus tailored to the values, experiences, and practices of community members [24, 35].

Guidance on engaging cancer survivors of color in patient-engaged research to address racial inequities in clinical trials is limited. To address this gap, we formed a community-academic partnership and present our experience establishing a Cancer Center Patient Advisory Council (PAC) at New England’s largest safety net hospital. The partnership included the Cancer Care Center at Boston Medical Center, Boston University School of Social Work Macro Department and the Center for Community Health Education Research and Service, Inc., a community-based organization focused on health equity and workforce development. The overall aim of this initiative was to: (1) recruit patients, family members, and/or caregivers, representative of the cancer care center’s patient population and (2) to develop a patient powered research agenda, whereby PAC members collectively identified targeted research priority areas.

## Methods

### Overview

We employed a community based participatory research approach to establish the PAC. Our goal was to recruit an 8–10 member PAC. Social networks both internal and external to the cancer care center were used to recruit PAC members reflecting the cancer care center’s majority African American, Black Caribbean, and Latinx patient population. We used an illustrative case study design to describe our integrative approach in establishing the PAC. All study protocols were approved by the Boston University Charles River Campus Institutional Review Board, protocol #4570X.

### PAC recruitment

The inclusion criteria for the PAC were self-identification as a cancer survivor (either undergoing active cancer treatment or a prior history of cancer), or a caregiver/family member of a cancer survivor, age 18 years or older, availability to meet monthly and willingness to commit as a member for at least one year. We developed flyers that outlined the main goals of the PAC which were to increase patient voice in cancer research and develop a strategic plan for patient-powered cancer research. PAC member roles would include participating in monthly trainings, designing and implementing activities to partner with cancer researchers, and giving formal presentations of their work. The flyer also described PAC membership as paid training (including transportation if needed and paid parking). Flyers were available in English, Spanish and Haitian Kreyol and distributed to cancer care center and primary care providers at Boston Medical Center and affiliated community health centers. Electronic mailings were also sent to providers, patient navigators, and the cancer care center support group director regarding plans and criteria to recruit members for the PAC.

To further enhance recruitment for the PAC and to educate the oncology academic community on the importance of patient engagement in research, the first and senior authors (M.C. & L.S.M.) co-presented at the Oncology Grand Rounds. They presented a framework on barriers to engaging patients of color in cancer clinical trials as well as potential solutions informed by their respective clinical practice and community engaged research [12, 15, 21]. Grand round attendees included clinical faculty, basic and translational science researchers, and clinical research staff. Clinical providers and staff were encouraged to discuss the opportunity to participate in the PAC with their patients. Recruitment for the PAC commenced in May 2017, with ongoing efforts to recruit new members on a rolling basis. After initial membership was established, for instance, PAC members were encouraged to recruit additional members within their individual social networks. All applicants interested in joining the PAC completed a brief application in writing or by phone with the research staff and was invited for an in person interview. The senior and second authors (L.S.M. and K.C.) conducted all PAC applicant interviews. Applicants completed a brief questionnaire either in writing or by phone with the research team and then participated in qualitative interviews. Interview questions elicited what potential members hoped to gain from being a patient advisory council member, skills or strengths they had to offer, previous experience with volunteer work, advocacy work, and research and their perceptions of the patient’s role in research. All interviews were audio recorded and verbatim transcripts were reviewed by the research team for content analysis.

### PAC training and evaluation

Once an initial cohort of 7 individuals confirmed interest in joining the PAC after the interview they underwent a group orientation and training program. PAC members worked with a facilitator (graduate research assistant) from the academic research team to develop a memorandum of understanding outlining their roles and responsibilities, as well as the duration of their term, which they were each asked to sign. If a council member decided to step down or was unable to fulfill their commitment, the PAC would nominate and vote on potential candidates to fill the empty seat.

**Figure 1:**
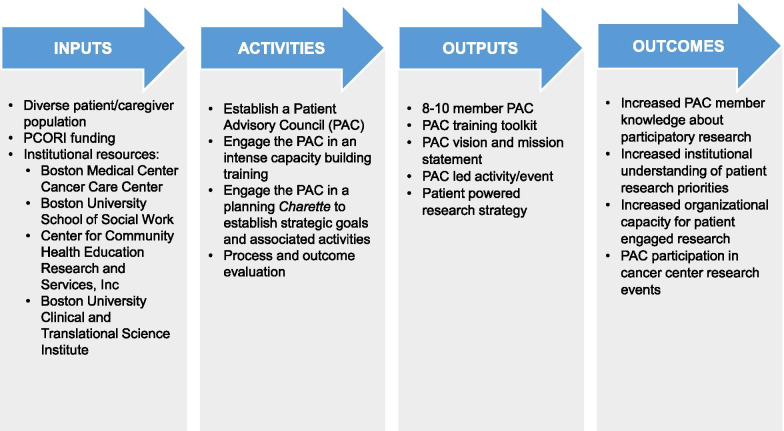
Patient advisory council training model to promote racial equity in clinical trials

PAC members participated in a four-hour orientation followed by capacity building training held at the Cancer Center which included an overview of the project’s aim to address racial inequities in cancer clinical trials, timeline, training approach and potential outcomes as illustrated in the training model (Fig. 1).

PAC members were also introduced to the concepts of community-based participatory research (CBPR) and patient-centered outcomes research (PCOR) [16, 36]. They received training on research ethics, institutional review boards and received didactic presentations on health equity, the social determinants of health, and community assessments. Content for the training was developed and facilitated by the project investigators and facilitators from the Boston University Clinical and Translational Science Institute Connecting Communities to Research Training Program [37]. As part of their training, PAC members also met with members of the Boston University Women’s Health Group patient advisory group to gain further insight on the experiences of another patient-centered research group.

To evaluate the process of establishing the PAC we used Tuckman’s stages of group development [38]. Key sources of data for this evaluation included responses to the PAC applicant interviews, direct observation of members during PAC meetings, document review from meeting minutes and agendas, as well as reports and materials generated by the PAC and academic research partners. Academic researchers used direct observation to document nominal group processes and to examine interpersonal interactions and participation during the PAC meetings. Academic researchers also took comprehensive notes and debriefed on their observations at the end of each meeting. Summary notes of these observations were analyzed thematically. PAC meeting agendas, materials presented during meetings, and meeting minutes were also reviewed. A comprehensive toolkit describing the project plan and materials was provided to the funding agency and is provided to demonstrate our process in detail (see Additional File 1).

## Results

### PAC recruitment

We received 18 referrals for the PAC from cancer care center patient navigators, oncology providers, and the cancer support group director. One of the referrals was from another applicant interested in joining the PAC. Of the 18 referrals, nine individuals were reached and seven applications were completed. Interviews were conducted in English (and Spanish as needed) and lasted between 15 and 20 min on average. All interviewed applicants were offered membership to the PAC. Demographics of the PAC included 5 females and 2 males, 4 identified as Black, 1 as Cape Verdean, 1 as Latinx and 1 as White. All members were proficient in English except one who exclusively spoke in Spanish. No PAC members had prior research experience. Two members reported experience with advocacy work outside of healthcare.

One applicant declined membership, concluding that participating would not be feasible with her treatment schedule and transportation needs, despite the research team’s efforts to facilitate transportation support. An additional member ultimately decided not to participate after attending one meeting, citing her caregiving responsibilities as an obstacle. PAC membership fluctuated over the course of the project, due to changes in PAC members’ availability—these challenges with recruitment and retainment are discussed below under *Lessons Learned*.

### Capacity building, goal setting, and action plan

We draw on *charrette* planning, a data driven iterative planning process in which one session informs the next, to engage the PAC in strategic planning for a patient powered research agenda [39]. PAC members engaged in critical discussions during monthly meetings integrating acquired knowledge from their research training with their own experience and/or perspective on racial inequities in cancer research to develop a strategic plan. The charrette planning process occurred for approximately 8 h over the span of 6 monthly meetings. Additionally, training resources from the Community Tool Box (an online curriculum) were used to support the PAC in developing their vision, mission, and strategic plan [40]. We used Tuckman’s four stages of small-group development: forming, storming, norming, performing and action planning [38] to describe and evaluate the process of establishing the PAC and their activities.

#### Forming

In the initial stage forming, group members’ central focus is orientation to the general purpose of the group and to each other [38]. PAC members described their own understanding of research processes in initial baseline interviews, which helped the research team further delineate existing strengths and areas for capacity building. The first two group meetings focused on orienting the PAC members to the purpose of the group and introducing them to the core concepts that would inform their decision-making on priority research questions. Orientation also included “icebreaker” and storytelling activities to build group cohesion and facilitate active engagement. Research team members introduced PAC members to the principles of patient engagement in research and CBPR, and provided essential background information on the cancer center and cancer care services at the hospital [16, 24]. We engaged PAC members in discussions on racial inequities in cancer care and cancer clinical trials participation.

Initial trainings involved a series of interactive presentations and group discussions. For instance, in the first meeting, the first author presented themes from a hospital-based study examining attitudes towards participating in clinical research among Black patients, and research team members facilitated a group discussion in response to the presented data. PAC members responded to several key themes around reticence to participate in medical research—such as “not wanting to feel like a guinea pig”—indicating the presence of similar concerns in their family networks or communities [12]. This sparked discussion of PAC members’ own positive and negative experiences as patients and caregivers. Additional training and capacity building activities within the first several meetings included a two-hour training session on research methods and ethics, and meeting with another hospital-based patient advisory group grounded in a CBPR approach. After the first and second meetings, a graduate assistant called PAC members individually to gather feedback. PAC members indicated enjoying getting to know each other through icebreaker activities, with a few expressing excitement at this opportunity to “give back” to other cancer patients and survivors. One PAC member described experiencing an “aha moment” during a presentation and discussion around racial health disparities, and members enjoyed hearing about another patient advisory council’s work. Collectively, feedback indicated that PAC members were developing a deepening understanding of the purpose of the PAC formation and the potential impact of patient advisory councils.

#### Storming

In storming, group members begin to establish mutual trust and become more comfortable voicing opinions, which may result in conflicts as group members learn how to work with each other [38]. PAC members began with varying levels of familiarity with cancer health disparities and how these disparities relate to clinical trials. As the PAC members became oriented to the core concepts relevant to the project over the course of the first two meetings, the focus in subsequent meetings shifted to engaging PAC members in active learning activities to prompt discussion and build knowledge. The research team used several strategies to facilitate learning and spark discussion on cancer health disparities and clinical trials, including discussion prompts such as word association activities, as well as the presentation of additional research studies and discussion of relevant topics in the media (such as an NPR article on racial disparities in clinical trials [41]. These learning opportunities prompted members to further reflect on their own experiences as patients and caregivers. For instance, the research team members asked PAC members to respond to the question: what do you think of when you think of clinical trials? A research team member listed PAC members responses out on poster board—a strategy used throughout the project to encourage brainstorming and create a shared visual record of the discussion in real time. This prompt led to a range of responses from PAC members—such as “*a study for beneficial outcomes*,” “*is this the best way to spend my time?*,” “*Mad scientist*,” “*What is the impact going to be on my body*?,” and “*can I get out of this once I start?*” Reflecting on their initial responses, PAC members began to share their initial thoughts and perspectives on barriers to cancer care and/or clinical trials, focusing primarily on a lack of trust in the medical system, within the Black community.

In this stage of group development, the authors continued to allocate a significant portion of each meeting to icebreaker activities designed to build group cohesion and mutual trust. The majority of the third and fourth PAC meetings focused on storytelling activities, with the research team members sharing their motivations for engaging in this research, carefully but intentionally touching on personal or family experiences of cancer, as well as professional experiences. PAC members shared their stories as cancer survivors or caregivers, and this process of mutual storytelling created a sense of shared experience and purpose. These team-building storytelling activities led to PAC members beginning to speak more openly with the academic-community researchers and each other, indicating an increasing sense of mutual trust and respect. In initial meetings, for instance, academic-community researchers noted that PAC members seemed reluctant to share perspectives or experiences that could be perceived as negative. By the fifth PAC meeting, about two months after the first meeting, they began to share a range of positive and negative experiences navigating healthcare systems, to ask pointed questions about the goals of the group, and to challenge each other to think critically.

#### Norming

As the group progresses to norming they have learned to function as a more cohesive unit invested in establishing their mutual goals [38]. In this stage, individual PAC members began to take greater leadership in facilitating meetings and action planning. This began gradually, with one PAC member, for instance, taking the lead in facilitating icebreaker activities, starting in the sixth meeting. Over the course of the first five meetings (about two months), PAC members had developed a working relationship with one another, and with academic-community research members, allowing them to begin the process of identifying and setting group goals. During meetings six through 11, spanning approximately four months, the PAC engaged in the process of developing a vision and mission and began identifying potential action steps for the PAC, using the VMOSA (vision, mission, objectives, strategies, action plan) model, a practical strategic planning tool [40]. A member from the academic research team presented on organizational visions and missions as key aspects of strategic planning, engaging the PAC members in evaluating different examples of visions and missions from healthcare and nonprofit organizations. The group spent several meetings brainstorming and unpacking individual members’ ideas, values and concerns relevant to the project’s core foci, which academic research members wrote out on poster board to create a shared visual record and facilitate further brainstorming. The group then worked together to identify shared or overlapping priorities, which involved significant back and forth between members. Academic research members and PAC members identified several recurring themes from the brainstorming sessions and PAC members’ discussions over the course of the group’s development. Themes included: the importance of community outreach and support; bolstering patient advocacy; improving patient-provider relationships, with a focus on trust, continuity and accountability; the significance of storytelling; and the need for a marketing “rebranding” of clinical trials. From these themes, the group worked to generate shared language to represent selected themes within a vision and mission. After several iterations, the PAC generated a vision:—“*Empowered patients in relationship with empathetic providers”* and mission: “*Advocating for patients to be in charge of their cancer care, supported by their doctor”* that they felt best represented their priorities and goals as a group.

#### Performing

In the final stage, performing, the group has established norms and identified each member’s role, and individual members take active roles in decision-making and planning [38]. Indicative of the final stage of group development, the PAC members now take active, leadership roles in group decision-making and action planning. Having developed a sense of group cohesion, working relationships based on trust and a shared vision and mission over the course of the last seven months,by the 12th meeting the group was able to focus solely on how to best advance their vision. PAC members voiced readiness to plan activities to move towards their group goals, and the research team supported this process by providing prompts to move the discussion forward. The PAC reviewed and considered their collective knowledge at this juncture, evaluating what they knew based on their personal, family and community experiences versus what remained unknown and of interest to them. Members reflected on the various strategies and ideas for action steps that had arisen during the process of developing a vision and mission, and concluded that the patient-provider relationship within cancer care had emerged as their primary area of shared interest. A few members expressed that without a trust-based patient-provider relationship, patient engagement in clinical trials was unlikely. The group decided that assessing other patients’ experiences with cancer care should be their first priority, and decided to develop and launch a patient assessment focused on patient and caregiver relationships with oncology and primary care providers. Over the course of the next year, the PAC members developed and conducted a patient assessment at the Cancer Care Center and analyzed the resulting data,with assistance from the academic research members. The results of this assessment and further details on this PAC-led process will be shared in a forthcoming paper, in collaboration with several PAC members.

## Lessons learned and discussion

Engaging patients, family members, and caregivers in research was an enriching experience for both PAC members as well as academic members of the community-academic partnership. We described the strategies employed to establish the PAC and to engage PAC members in developing a patient powered research agenda at a safety net cancer care center. Our focus on the process guided by the integration of frameworks for a CBPR approach and group development led to the successful establishment of a PAC representative of a safety net hospital’s patient population. To our knowledge, this integrated approach to building capacity for a hardly reached patient population impacted by cancer to engage in research to address racial inequities in clinical trials has not been previously described.

One of the key lessons was intentionality in recruiting patients of color, particularly Black patients and caregivers. Although prior literature described challenges in recruiting patients reflecting the demographics of the health system or diverse patient populations to patient advisory councils [26, 27], we were able to engage Black patients and caregivers through our focused efforts at a safety net hospital system serving a large population of patients of color. Our focused approach to recruitment and ability to engage individuals without prior research experience adds to the literature strategies that can help diversify the narrative of patients with cancer and caregivers engaged in research.

Integrating the lived experiences of patients and caregivers with capacity building activities empowered the PAC to advocate for themselves and others. The PAC acquired evidence based knowledge on racial disparities in cancer care and cancer outcomes in addition to skills training in participatory research methods. PAC members integrated the knowledge gained with their own lived experiences to develop a vision and mission statement that illustrated their collective priority for promoting patient autonomy and empowerment. Moreover, PAC members used this engagement initiative to advocate for themselves even beyond the scope of patient engaged cancer research. One member reported applying her newly acquired skills to obtain employment in the medical field and another member decided to further enhance her computer literacy skills by enrolling in a computer training course.

Patient-engaged research takes time for both establishing trust and getting members ready to engage as research partners. Taking several months to establish trust among PAC members and between PAC members and the academic research team was necessary to engage the PAC in critical discussions for developing a patient and caregiver led research agenda. Setting ground rules at the outset that every voice matters and that every member has something to contribute engendered this trust and openness to the process. This process also led to power sharing and co-learning, a key feature of CBPR, among PAC members and between PAC members and the academic research team [24, 29]. Early in the stage of PAC group development, members were informed of their role to ultimately lead the patient engagement initiative. Academic research members exercised patience in this process, giving the PAC members the time needed for growth into their leadership position.

Time was also a critical factor in the pace of the PAC’s decision-making process. CBPR is often slower than investigator-driven research because it requires that the community involved in the work (in this case the PAC) not only contribute to the work but actually develop the research agenda. One of the aims centering people of color in patient-engaged research was to empower patients with the knowledge and capacity to establish a patient powered research agenda at Boston Medical Center Cancer Care Center. The themes that emerged from the charrette focused on the relationship and communication between patients and cancer care providers which set the stage for development of the assessment administered to the patients and caregivers at the cancer care clinic.

Academic researchers gained a deeper appreciation for the PAC’s perspectives on approaching the problem of racial inequities in clinical trial participation as this generated new ideas and approaches to the problem. Based on the presented literature, academic researchers anticipated that the PAC would consider a patient centered educational or awareness campaign for participating in cancer clinical trials, but PAC members astutely identified patient empowerment and focusing on patient-provider interactions as the key priory. Academic researchers additionally witnessed the transformative nature of patient-engaged research. PAC members initially participated in the Cancer Center’s health disparities conferences as attendees but later one PAC member gave an oral presentation along with the academic research member L.S.M. on “*Interdisciplinary collaboration and community engagement as tools for tackling inequity: Establishing a mechanism for patient powered cancer research at a safety-net hospital.*” at the 2018 annual American Public Health Association meeting. PAC members have also presented initial findings from their patient and caregiver assessement to the Cancer Center transdisciplinary clinical and research leadership team (manuscript is forthcoming) and have provided guidance on curriculum development for a community partner project and a multi-site cancer center grant.

There were a few challenges that emerged during the course of establishing the PAC. Member retention and new member recruitment were challenging. The advisory council had a total of seven members, with five members consistently engaging in PAC activities for most of the 2-year project. Two PAC members, one active in the PAC for approximately one year and the other approximately 6 months, returned to work. One initial member was the principal caregiver for her mother and was primarily comfortable speaking in Spanish. A bilingual academic research team member provided translation but being the sole member not fluent in English may have contributed to her early withdrawal from the group. Additional obstacles to long-term participation arose due to the endemic nature of working with a population with active pressing health needs. One member undergoing active cancer treatment actively participated in PAC meetings for the first year, after which team members were no longer able to reach him, despite repeated attempts at contact.

Identifying potential members with the availability to commit to one year was a challenge and was pursued through multiple channels including referrals from current group members and community-based organizations. Given the uncertainty of the clinical status or follow up appointment burden for individuals living with cancer and their caregivers we wanted to incorporate flexibility in the time commitment to participate in the PAC. PAC members did identify potential strategies for recruiting additional members including a plan to engage with patients/caregivers that expressed interest in learning more about the council while conducting their patient assessment. They also considered combining membership with another hospital patient advisory group. Unfortunately, the COVID-19 pandemic did halt further in person recruitment efforts. Virtual conferencing has been used to connect with active PAC members but the research team is mindful of the potential digital divide and exclusion of potential members without internet capabilities or comfort with using the internet.

Another challenge was organizational buy-in to sustain the patient advisory council. Cancer center clinical and nonclinical researchers had varying familiarity with patient engaged research [42] and thus had not considered how to financially sustain the PAC beyond the funded 2-year project. The PAC members and principle investigators gave a presentation to cancer center leaders at their quarterly meeting and are currently in negotiations in integrating the PAC within hospital and cancer center operations.

Despite the challenges, our interdisciplinary community engaged team was successful in recruiting and retaining a diverse PAC over time. Attention to group dynamics and relationship development allowed PAC members to establish relationships with researchers, graduate students and community partners. Relationship development may have also been enhanced by the concordance between the PAC and the research team, with researchers, community partners and students identifying as Black, Haitian-American, Latinx and Lebanese.

## Conclusions

In summary, we present our strategy in establishing a patient advisory council to develop a patient powered research agenda among Black individuals and other patients and caregivers traditionally underrepresented in cancer research. We described our capacity building strategy to promote patient powered research. Although generalizability of this approach is limited given implementation at a single institution, this work demonstrates the transformative power of meeting patients where they are at, relationship-building, shared power and co-learning. Patients’ lived experiences coupled with our community-academic partnership ultimately led to the design and implementation of a PAC-conducted needs assessment at a safety net cancer care setting. This process shifted patient perceptions of their ability to contribute to change in cancer care and provided a platform for centering their lived experiences in cancer research. Sustainability of the PAC will require organizational commitment and continued financial support.

## Supplementary Information

**Additional file 1.** Connecting Hardly Reached Patients to Research Toolkit.

## Data Availability

Not applicable.

## Abbreviations

CBPR: Community-based participatory research
PAC: Patient of Advisory Council
PCOR: Patient-centered outcomes research
VMOSA: Vision, mission, objectives, strategies, action plan
