# Supplementary material for: Patient powered research: an approach to building capacity for a hardly reached patient population to engage in cancer research
Source: Res Involv Engagem. 2021 Oct 26;7:74. doi: 10.1186/s40900-021-00317-7 (PMC8547568; doi:10.1186/s40900-021-00317-7)
Supplement: Supplementary file 1 — Additional file 1. Connecting Hardly Reached Patients to Research Toolkit. [file 40900_2021_317_MOESM1_ESM.pdf]

INTRODUCTION

Community engagement can provide a more nuanced understanding of patient experiences, priorities and community conditions, while simultaneously empowering communities. A patient advisory council (PAC) is a method of community engagement that can be used in effectively in clinical research settings. This guide is designed to assist healthcare researchers and providers who are interested in establishing a PAC. The following documents are included in the guide:

1. Project description: Patient Powered Cancer Research at a Safety Net Hospital
2. About the PAC at the Cancer Center at Boston Medical Center
3. Sample PAC application form
4. Sample PAC establishment procedure
5. Sample PAC dissemination plan
6. Diagram of PAC establishment process

BU Boston University School of Social Work

BOSTON MEDICAL CENTER
EXCEPTIONAL CARE. WITHOUT EXCEPTION.

CHERS Center for Community Health Education Research and Service, Inc.

Patient-Powered Cancer Research at a Safety Net Hospital

About the project:

Boston is home to some of the most premier health care facilities and academic institutions in the world. However, not all Bostonians benefit from the health care innovations the city is known for. Black Bostonians experience a disproportionate burden of chronic conditions, including cancer. **Despite higher rates of cancer-related morbidity and mortality, nationally, Blacks are far less likely than their White counterparts to enroll in clinical trials and to receive cutting edge treatments.**

Community engagement can provide researchers with a more nuanced understanding of patient experiences, priorities and community conditions, while simultaneously empowering communities.
Engaging community stakeholders in the research process, from planning and implementation to dissemination, may also facilitate translational research efforts by creating a common way of talking about and understanding research, deciphering the science so that it can be incorporated into practice settings.

Currently there is not a mechanism within the Cancer Center by which patients can connect with research, and inform PCOR activities. **Through community engagement, we seek to catalyze research innovation and increase both survivor and provider knowledge establishing a Cancer Center patient research advisory council (PAC).** The PAC will work with cancer center researchers to build a patient powered research agenda whereby cancer survivors and their family members will collectively identify research priority areas, contributing to the overall vision of the cancer center.

Project partners:

The Cancer Center at Boston Medical Center is dedicated to providing accessible healthcare to everyone. The Center's experienced cancer specialists combine their extensive knowledge and expert skills to focus on each patient's needs.

BOSTON MEDICAL CENTER
EXCEPTIONAL CARE. WITHOUT EXCEPTION.

Boston University School of Social Work has an urban mission to advance a just and compassionate society that promotes health and well-being and the empowerment of all oppressed groups, especially those affected by racial, social, and economic inequities.

BU
Boston University School of Social Work

The Center for Community Health Education, Research, and Service, Inc. was established in 1991 among Boston Medical Center, the Boston Public Health Commission, Boston University School of Medicine, Northeastern University Bouvé College of Health Sciences and an established network of fifteen community health centers (CHCs) serving the racially and ethnically diverse populations in the central city neighborhoods of Boston.

CHERS Center for Community Health Education Research and Service, Inc.

Project Contact:

Marjory Charlot, MD, MPH, MSc
617.638.6428

Linda Sprague Martinez, PhD
617.358.0782

Elmer Freeman, MSW
617.373.5179

About the Patient Advisory Council

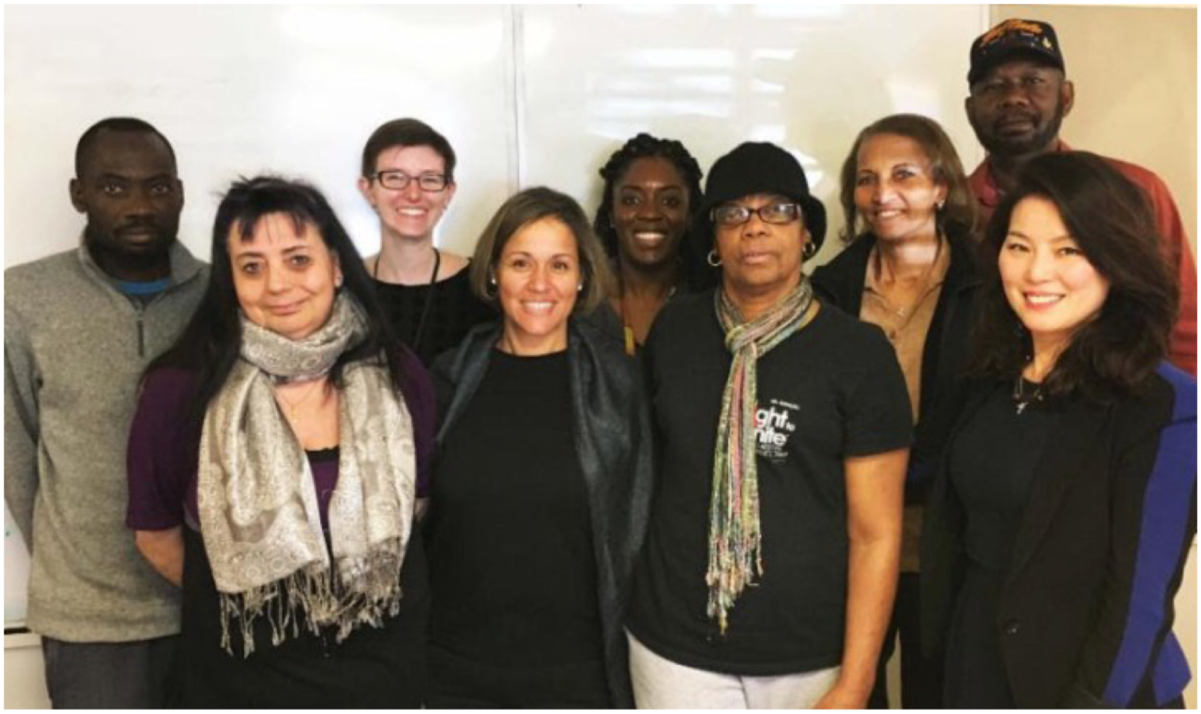

Pictured: PAC members Dirisa Mawanda, Judy Murphy, Cheri Clark, Donnetta Andrews, and Joseph Jones with Nicole Spencer and Jennifer Pamphile of the Center of Excellence in Women's Health at the Boston University Medical Campus, and Dr. Linda Sprague Martinez and Dr. Hyeouk "Chris" Hahm of Boston University School of Social Work

Vision Statement:

Empowered patients in relationship with empathetic providers.

Members of the PAC emphasize the importance of communication between providers and patients. Providers and patients should be on the same page when it comes to expectations for and understanding of care. Patients should be able to express their needs, concerns, and priorities to their provider, and providers should be ready to explore and address these needs, concerns and priorities.

Mission Statement:

Advocating for patients to be in charge of their cancer care, supported by their doctors.

Boston University School of Social Work

Boston Medical Center — Exceptional Care. Without Exception.

Center for Community Health Education Research and Service, Inc.

Patient Advisory Council Member Application

NAME:____

ADDRESS:________________________ CITY:______________ STATE: MA ZIP:__________

HOME PHONE:_____________ CELL PHONE:_____________

EMAIL: _____________________ DATE OF BIRTH:____/____/____

Please answer the following questions (circle yes or no):

1. Are you at least 18 years of age?	YES NO
2. Are you a Boston Medical Center patient?	YES NO
3. Can you commit to the position and the project for at least 1 year?	YES NO
4. Are you available monthly on Tuesdays from 10 am-12pm?	YES NO
5. Are you a cancer survivor?	YES NO
6. Do you have previous experience with research, grass-roots action, coalitions, or advocacy work?	YES NO
7. Are you willing to learn and be a part of a team?	YES NO

Please answer the following 5 short answer questions:

1. How did you hear about this position?

2. What do you hope to gain from being a member of the board?

3. What skills and/or strengths would you bring to the advisory board?

4. Have you ever done any volunteer work in your community? For example, have you ever helped a neighbor, volunteered with your church or mosque, tutored another student, helped at a soup kitchen, etc.? Explain.

5. Describe any previous experience with research, grass-roots action coalitions, advocacy work, and/or any previous experience in health or public health:

BU
Boston University School of Social Work

BOSTON MEDICAL CENTER
EXCEPTIONAL CARE. WITHOUT EXCEPTION.

CHERS
Center for Community Health Education Research and Service, Inc.

Establishment of the Patient Advisory Council

Group Development Stages and Activities:

Forming- PAC members come together and get to know one another through various icebreakers and storytelling activities.
Storming- PAC members participate in capacity building activities, learn more about cancer clinical trials via articles and NPR pieces, start to ask pointed questions about goals of the group and challenge one another to think more critically.
Norming- PAC members develop a shared vision and a working relationship with one another. As they begin goal setting, they start to take over more planning responsibilities.
Performing- PAC members take an active role in decision making and planning, they have an agenda and begin to plan activities to move their work forward.

**Tuckman, B.W. & Jensen, M. A. C. (1977). Stages of small-group development revisited. Group & Organization Management, 2(4), 419-427.

Capacity Building

Knowledge of Cancer and Clinical Trials:

PAC members began with varying levels of understanding around cancer health disparities and how they related to clinical trials. Through various learning opportunities, including pieces from the Boston Globe, NPR, and research pulled together by project investigators, PAC members have gained more knowledge on both topics. Furthermore, this learning process has helped to improve members' ability to describe and reflect on their own experiences as patients and caregivers.

Community Role in Research:

PAC members detailed their own experiences with and understanding of research processes in initial informational interviews. After a few meetings, the group also had the opportunity to meet and learn from another community based participatory research model: The Patient Advisory Group at Boston Medical Center. In addition, all members participated in a two-hour training session on research methods. Members will use what they have learned to guide their own research topics.

Developing Vision and Action Plan:

After several meetings as a group, themes such as the importance of storytelling and the need for informed providers began to emerge. From these themes, the PAC developed a vision and mission using a VMOSA Model. The group then began to develop an action plan based on the chosen vision and mission.

Patient Advisory Council Dissemination Plan

Audience

The Patient Advisory Council plans to present their findings and materials to important stakeholders in improving cancer care.

Providers	Patients	Administration	Support
Oncologists	Cancer patients	Leadership	Caregivers
Primary Care Physicians	Other patients	Management	Family
Nurse Practitioners			
Surgeons			
Social Workers			
Care Coordinators/Navigators			

Outlets

Materials produced by the Patient Advisory Council will be distributed through multiple outlets to reach as large an audience as possible.

Oral Presentations	Multimedia	Mass Media	Other
Grand rounds	Videos	Radio stations	Kick-off events
Staff and team meetings	Newsletters	Newspapers	Contests
Patient groups at community health centers and in the hospital	Emails to survey participants	Online news outlets	
Case consultations	Posters/flyers		
Workshops	Social media		
	Blogs		

Materials

The Patient Advisory Council aims to develop products that will target all stakeholders. These materials will be centered around the results of the survey that the Council has developed to assess the experiences of patients receiving care at Boston Medical Center. Data collection is currently in process.

BU Boston University School of Social Work

Boston Medical Center — Exceptional Care. Without Exception.

CHERS Center for Community Health Education Research and Service, Inc.

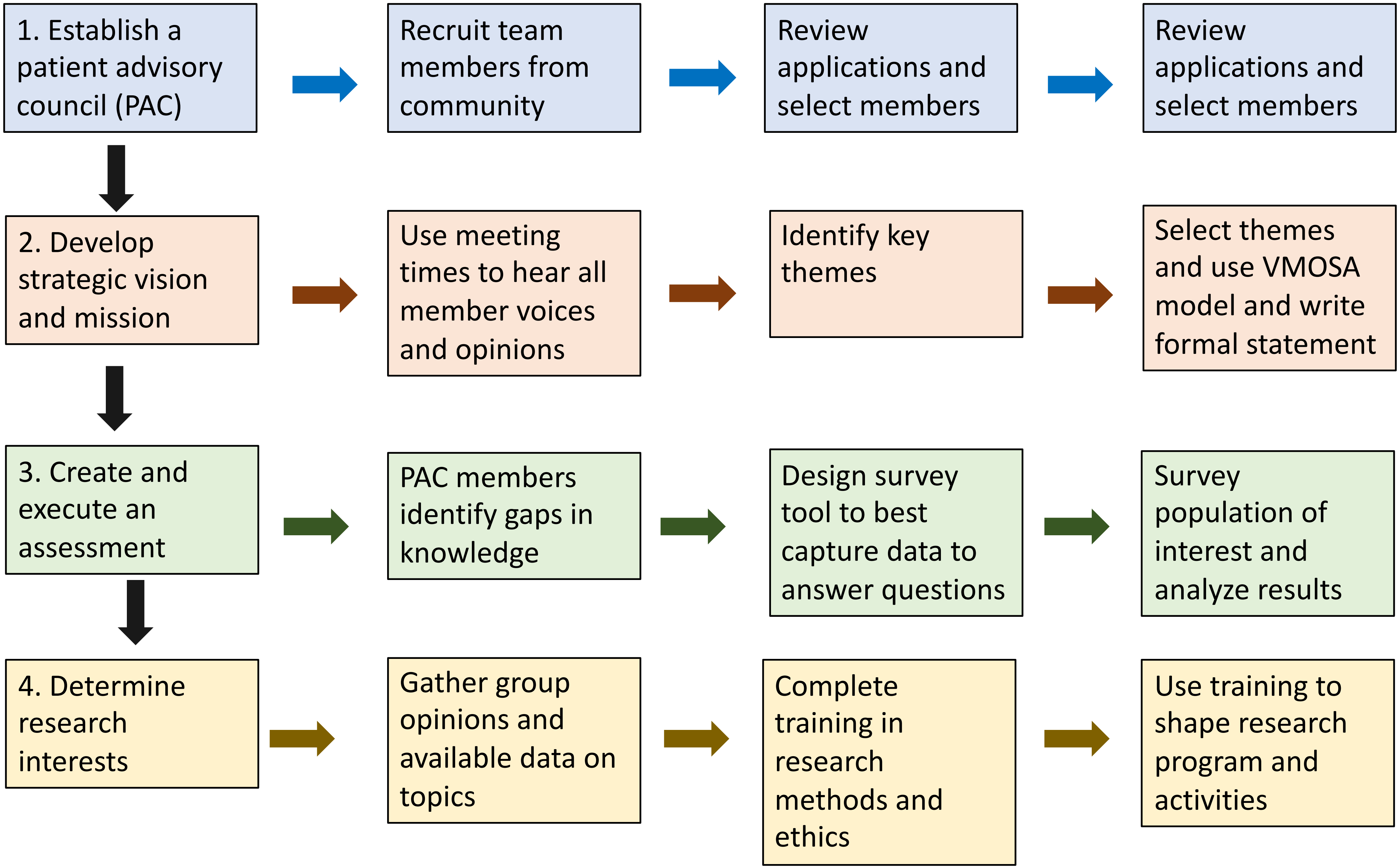
1. Establish a patient advisory council (PAC)
Recruit team members from community
Review applications and select members
Review applications and select members
2. Develop strategic vision and mission
Use meeting times to hear all member voices and opinions
Identify key themes
Select themes and use VMOSA model and write formal statement
3. Create and execute an assessment
PAC members identify gaps in knowledge
Design survey tool to best capture data to answer questions
Survey population of interest and analyze results
4. Determine research interests
Gather group opinions and available data on topics
Complete training in research methods and ethics
Use training to shape research program and activities
